# Exploiting intraspecific competitive mechanisms to control invasive cane toads (*Rhinella marina*)

**DOI:** 10.1098/rspb.2012.0821

**Published:** 2012-06-13

**Authors:** Michael R. Crossland, Takashi Haramura, Angela A. Salim, Robert J. Capon, Richard Shine

**Affiliations:** 1 School of Biological Sciences A08, University of Sydney, New South Wales 2006, Australia; 2 Division of Chemistry and Structural Biology, Institute for Molecular Bioscience, University of Queensland, Queensland 4072, Australia

**Keywords:** alien species, anuran larvae, biocontrol, *Bufo marinus*, wildlife management

## Abstract

If invasive species use chemical weapons to suppress the viability of conspecifics, we may be able to exploit those species-specific chemical cues for selective control of the invader. Cane toads (*Rhinella marina*) are spreading through tropical Australia, with negative effects on native species. The tadpoles of cane toads eliminate intraspecific competitors by locating and consuming newly laid eggs. Our laboratory trials show that tadpoles find those eggs by searching for the powerful bufadienolide toxins (especially, bufogenins) that toads use to deter predators. Using those toxins as bait, funnel-traps placed in natural waterbodies achieved near-complete eradication of cane toad tadpoles with minimal collateral damage (because most native (non-target) species are repelled by the toads' toxins). More generally, communication systems that have evolved for intraspecific conflict provide novel opportunities for invasive-species control.

## 1. Introduction

Most examples of successful biocontrol of invasive taxa rely upon exploiting interspecific interactions, by bringing in species-specific pathogens [1,2], predators [3] or herbivores [4], often from the invader's native range. There have been surprisingly few attempts to exploit intraspecific interactions in the same way. For many species, the greatest threat to an individual's viability comes from conspecifics rather than heterospecifics, resulting in the evolution of complex and sophisticated systems for intraspecific conflict [5–8]. This species-specificity of intraspecific communication systems creates opportunities for targeted control of the invader with minimal effects on native taxa, especially in cases where the invader is phylogenetically distant from the local biota [9–13].

The rapid spread of cane toads (*Rhinella marina*; formerly known as *Bufo marinus*) through tropical Australia has been devastating for native predators (including fishes, frogs, lizards, snakes, crocodiles and marsupials) that are poisoned when they attempt to eat these toxic newcomers [14–16]. That impact has stimulated vigorous attempts to control toad numbers, mostly via capture and removal of toads during the terrestrial phase of their life history [17]. Unfortunately, the toad invasion has continued unabated [18].

Sophisticated pheromonal communication systems in cane toad larvae provide opportunities for control. Intense competition within the pond environment means that older toad tadpoles benefit from reducing the numbers of freshly laid eggs [19–21]; and thus, the toad tadpoles actively search out and consume such eggs, based on waterborne cues [22]. If we can identify the specific chemical(s) attracting the cannibal's attention, we could remove toad tadpoles from a waterbody by using those chemicals as bait in funnel-traps [22].

We conducted laboratory trials to fractionate materials produced by toad eggs and to quantify the responses of toad tadpoles to those chemicals. We identified the toads' distinctive chemical defences (bufadienolides) as a powerful attractant for toad tadpoles (and a repellent for native species), and then conducted field trials to assess the feasibility of using these toxins as bait in traps to selectively eliminate toad tadpoles from natural waterbodies.

## 2. Material and methods

Field observations of toad tadpoles being attracted to newly laid clutches of toad eggs suggested that the egg mass produces some attractant substance [23]. Laboratory trials confirmed that prediction [22], and so we proceeded to fractionate egg-mass exudates in an attempt to identify biologically active compounds.

Toad eggs from laboratory-laid clutches were freeze-dried, extracted with methanol and dried *in vacuo*. The crude methanol extract was partitioned between butanol and water. The butanol soluble extract was active in the attractant bioassay and was defatted with *n-*hexane. The defatted butanol extract was fractionated with semi-preparative high-performance liquid chromatography (HPLC, Agilent Zorbax SB-C8 column, 5 μm, 9.4 × 250 mm, eluting from 90% water/acetonitrile to 100% acetonitrile in 15 min, wash in 100% acetonitrile for 12 min, 3.5 ml min^−1^) to give 26 fractions.

These fractions were presented to groups of 20 toad tadpoles (midway through development, at Gosner [24] stages 30–38) in standardized trials to measure attraction and feeding responses (for methods of collection, husbandry and testing, see [21]). All toad tadpoles came from the toad population on the Adelaide River floodplain, 60 km east of Darwin, in the Northern Territory. At 5 min intervals after the stimulus was presented (1 ml dropped onto the water surface inside a mesh box in one corner of a 70 × 45 × 9 cm plastic tray, with water 5 cm deep), we scored the number of toad tadpoles within the quarter of the tray closest to the stimulus origin, and the number that were actively feeding (head-down posture, tail wriggling). Simultaneously conducted control trials allowed us to test the statistical significance of any behavioural responses to the stimuli presented.

**Figure 1.**
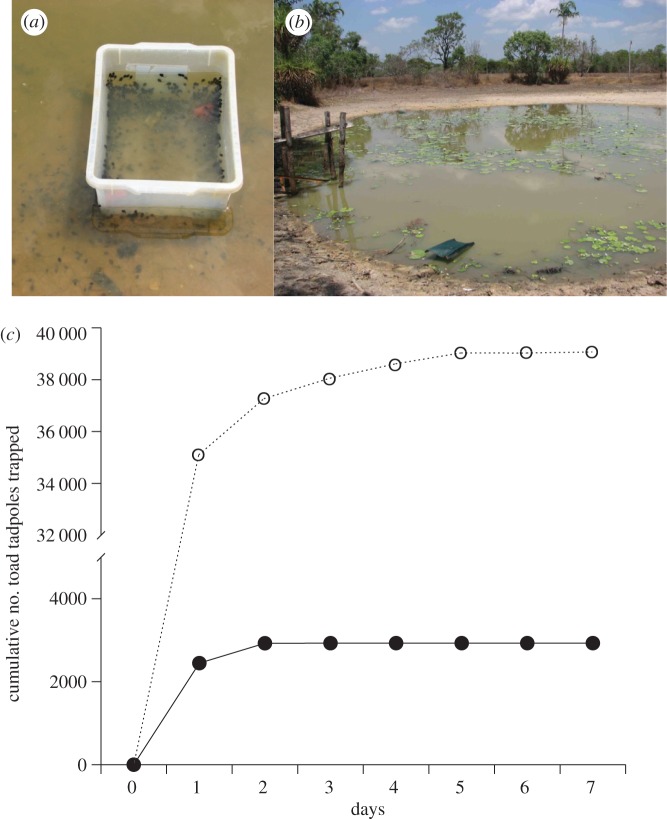
Effectiveness of toxin-baited toad traps in the field. (*a*) Funnel-trap in pond 2, showing funnels (red objects); note toad tadpoles attempting to enter trap. (*b*) Funnel-traps deployed at pond 1, under shadecloth. (*c*) Cumulative numbers of tadpoles of the invasive cane toad (*Rhinella marina*) caught in two natural waterbodies in tropical Australia, using funnel-traps baited with exudate from the parotoid glands of adult cane toads. Filled circles with solid line, pond 1; open circles with dashed line, pond 2.

We made funnel-traps (figure 1*a,b*) from rectangular plastic boxes with holes cut on two diagonally opposite sides to accommodate plastic funnels (holes 6.5 cm diameter; funnel length 5 cm, minimum internal funnel diameter 13 mm). To obtain toxin for laboratory and field trials, we held an adult toad in gloved hands beneath a protective sheet of glass, and gently squeezed the shoulder (parotoid) glands to expel the toxin onto the underside of the glass. For field trials, the exudate was wiped off onto a glass microscope slide and weighed (2 ± 0.1 g per slide, requiring about four toads) and one slide per funnel-trap was used as bait, replaced daily. The quantity of toxin used was based on pilot studies in wading pools.

Six traps were placed at equal intervals around the margins of each of two natural waterbodies on the Adelaide River floodplain (pond 1 = 84 m perimeter, 12°34′39″ S, 131°19′4″ E; pond 2 = 78 m perimeter, 12°45′6″ S, 131°29′8″ E). Traps were placed in shallow water (13 cm), with shadecloth covers to prevent overheating at midday. On the morning of day 1, we added the chemical bait. Traps were then checked and cleared every 24 h for the next 7 days; all trapped individuals were removed and held in captivity to prevent them being re-captured. If toad tadpoles were too numerous to count by hand, we weighed 100 representative specimens and estimated capture rates based on total mass divided by mean mass per toad tadpole. We also conducted daily (diurnal and nocturnal) visual surveys for toad tadpoles, metamorph cane toads and fishes before, during and after the trapping sessions.

Because the ponds we trapped did not contain tadpoles of native frogs, we conducted additional laboratory trials to clarify the species-specificity of that attractant response. A funnel trap containing 1 g of parotoid exudate (obtained from two to four toads, as above) on a glass slide was placed in a large circular wading pool (2.4 m diameter, 15 cm deep, 700 l) containing 100 tadpoles either of cane toads, or of native species (either *Litoria bicolor, Litoria caerulea, Litoria nasuta, Litoria rubella* or *Litoria rothii,* all obtained by collecting egg masses in local waterbodies, and raising tadpoles in captivity until testing). An adjacent pool containing an identical but unbaited funnel-trap (i.e. containing a glass slide without toxin) served as a control. Two replicates were run for treatment and control for each of the six species (i.e. cane toads and the five treefrogs). In each case, we scored the numbers of tadpoles trapped within 24 h after the trial commenced.

## 3. Results

**Figure 2.**
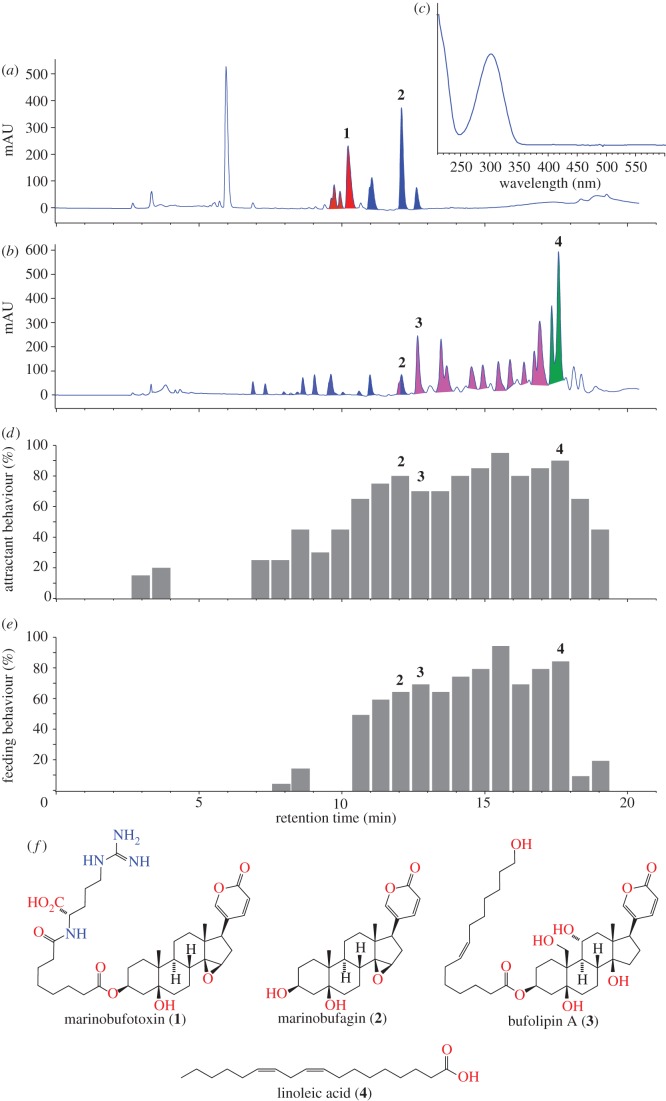
Chemical composition and biological effects of cane toad eggs. Chemical analysis (HPLC-DAD) of (*a*) cane toad parotoid secretion and (*b*) eggs. (*c*) Bufadienolide UV-vis spectrum. (*d*) Attractant and (*e*) feeding behaviour assay results on fractionated egg extract. (*f*) Representative examples of bufotoxins (**1**), bufogenins (**2**), bufolipins (**3**) and essential fatty acids (**4**) isolated from cane toad parotoid secretions and/or eggs. Red regions, bufotoxins; blue regions, bufogenins; pink regions, bufolipins; green regions, essential fatty acids.

Preliminary analysis revealed a higher chemical diversity in cane toad eggs than in cane toad parotoid secretion (figure 2*a*,*b*). HPLC-diode-array detection (DAD) analysis confirmed that both egg and glandular secretions were dominated by bufadienolides, a class of steroidal Na+/K+ ATPase inhibitors possessing a diagnostic ultraviolet (UV)-vis chromophore (figure 2*c*), while mass spectrometric analysis revealed three distinct classes: bufotoxins, bufogenins and bufolipins (**1**–**3** in figure 2*f*). Whereas cane toad parotoid secretion was dominated by bufogenin **2** with trace levels of the highly nitrogenous bufotoxin **1** (figure 2*a*), the eggs were rich in bufogenin **2** and bufolipin **3**, as well as ‘essential fatty acids’ **4** (figure 2*b*). Pure samples of **1**–**4** (figure 2*f*) isolated from secretions and/or eggs were characterized by detailed spectroscopic analysis (see the electronic supplementary material), and used as authentic standards for chromatographic comparisons.

As previously reported for eggs of the toad *Bufo arenarum* [25], the eggs of cane toads contained the essential fatty acid linoleic acid (**4** in figure 2*f*). In contrast to essential fatty acids, bufadienolides have a far more restricted distribution [26]. For example, in Australia bufadienolides are produced by only two species, the invasive pest animal *Rhinella marina* (the cane toad) and the invasive weed *Bryophyllum tubiflorum* (mother of millions [26,27]). In cane toads, bufadienolides have been detected in all phases of the life cycle [28] and are a major contributor to cane toad toxicity. Cane toad parotoid glands secrete high levels of cardiotoxic bufotoxins and bufogenins [29], whereas the skin contains bufotoxins [30] and dissected ovaries/eggs contain bufogenins [31] and bufolipins [32]. If ingested, these toxins are fatal to many Australian animals that have not been exposed to bufadienolides over evolutionary time [16].

To explore the potential for cane toad egg metabolites to drive intraspecific chemical communication, we fractionated egg extracts and subjected these fractions to behavioural assays to detect toad tadpole attractant and feeding responses. These studies localized behavioural effectors in fractions rich in bufogenins, bufolipins and fatty acids (figure 2*d*,*e*; statistical tests (results not presented) show that all cases with greater than 50% of tadpoles exhibiting either attraction or feeding were significantly (*p* < 0.05) different from control trials, even after Bonferroni correction). In our tests of the pure compounds, bufotoxin **1** did not elicit any significant behavioural response, whereas bufogenin **2** and bufolipin **3** stimulated both attractant and feeding activity. Pure linoleic acid **4** did not elicit any attractant or feeding response, suggesting that the apparent attractivity elicited by the egg fraction was owing to trace amounts of co-eluting bufolipins (as supported by nuclear magnetic resonance analysis).

Some of the substances present in the toad egg would be likely to attract native predators as well, and thus would not provide targeted control (i.e. would attract too many non-toad species to traps). However, toad tadpoles responded strongly to toad-specific toxins (bufadienolides) that are known to repel native tadpoles and fishes [33]. Those contrasting responses provide an opportunity for toad-specific control. Although there is little overlap in the bufadienolide contents of the parotoid secretion and egg, at least two common bufogenins (telocinobufagin and marinobufagin) are present in both materials, both of which were active in the attractant behavioural assay. Importantly, the parotoid secretions of adult cane toads contain high levels of bufogenins (figure 2*a*). The toad parotoid secretion is easy to obtain (adult cane toads are readily found, collected and ‘milked’), whereas eggs are available only seasonally, persist for only a day or two after oviposition, and hence are difficult to collect in sufficient quantities for use in landscape-scale toad control. Thus, the parotoid secretions offer a more readily accessible source of bufogenins than do egg extracts.

In our field trials, funnel traps baited with toad toxin (exudate from the parotoid glands of adult cane toads) rapidly caught tens of thousands of toad tadpoles, and very little else (figure 1). The ponds contained thousands of native fishes (greater than 5000 per pond, based on visual surveys before and after our trapping sessions) but the toxin-baited traps caught less than 30 fish (all trout gudgeons, *Mogurnda mogurnda*). Thus, vertebrate bycatch constituted less than 0.1 per cent of animals caught (42 000 toad tadpoles, 27 fish). The traps also captured 24 invertebrates, mostly water scorpions and beetles (both present in each pond in thousands). The rapid reduction (to zero) in capture rates (figure 1*c*) suggests that we caught most toad tadpoles in the ponds. Because metamorph toads are diurnal and are restricted to pond margins from the time of emergence until rain falls, the numbers of recruiting metamorphs can be accurately determined by visual counts [34]. Our surveys detected no metamorph toads emerging from the ponds in the two weeks following trapping sessions.

Our wading-pool trials showed that the parotoid secretions of adult cane toads attracted toad tadpoles into traps, but repelled the tadpoles of native frog species. The numbers of toad tadpoles captured in toxin-baited traps was higher than in empty (control) traps (totals 186 versus 41; against a null of equal numbers, **χ**^2^_1_ = 92.62, *p* < 0.001) whereas the reverse was true for tadpoles of *Litoria nasuta* (16 versus 40, **χ**^2^_1_ = 10.29, *p* < 0.01), *L. rothii* (53 versus 117; **χ**^2^_1_ = 24.09, *p* < 0.001) and *L. rubella* (39 versus 106; **χ**^2^_1_ = 30.96, *p* < 0.001). Parotoid-baited and control traps caught similar numbers of tadpoles in trials with *L. bicolor* (13 versus 7; **χ**^2^_1_ = 1.80, *p* > 0.15) and *L. caerulea* (39 versus 57; **χ**^2^_1_ = 3.38, *p* = 0.06).

We replenished bait daily in our field trials, but laboratory studies suggest that baits can remain effective for at least 3 days: capture rates per 100 tadpoles per day in large wading pools fell from 93 per cent for fresh parotoid secretion, to 69 per cent for 1-day-old secretion (kept in water throughout the intervening period), to 54 per cent for 2-day-old secretion, to 45 per cent for 3-day-old secretion.

## 4. Discussion

Our results suggest a new way to control an invasive species that is causing catastrophic ecological damage in Australia. To locate freshly laid conspecific eggs, the tadpoles of cane toads use waterborne cues that include the toads' own chemical defences (bufadienolides: figure 2*b*,*f*). By consuming conspecific eggs, older toad tadpoles reduce the number of future competitors, and also obtain nutrition and possibly, toxins [22]. Toad tadpoles also frequently cannibalize dead adult toads in waterbodies, and the toxins in those dead adults may well be the attractant that stimulates that behaviour. The toad tadpoles' ability to detect conspecific toxins, and their intense attraction to those toxins, enabled us to remove most or all toad tadpoles from natural waterbodies with a few days' trapping (figure 1).

Although our trials targeted the ‘cannibal attractant’ response, toad tadpoles also produce and respond to chemicals in other contexts. For example, stressed and injured toad tadpoles produce alarm chemicals that induce rapid escape reactions in conspecifics and inhibit tadpole survival, growth and development [35]. Toad tadpole viability is similarly reduced by short-term exposure of the eggs to chemical cues from older toad tadpoles [36]. A better understanding of the chemical nature of those cues might well facilitate other toad-control methods.

One of the most important issues for any invasive-species control programme is to avoid collateral damage; that is, the control efforts should affect the invader only, not native taxa. This aim can be difficult to achieve: for example, an inability to identify cane toads has resulted in much inadvertent mortality of native frogs [37]. Critically for field implementation, our funnel traps caused minimal collateral damage: toad tadpoles comprised greater than 99 per cent of vertebrates trapped, and greater than 98 per cent of all animals trapped. Toad tadpoles are among the smallest aquatic vertebrates (compared with fishes, and the tadpoles of most native frogs), allowing the use of funnel traps with apertures too small (13 mm) to allow ingress by most non-target taxa. More importantly, however, cane toad toxins are detected and avoided by native tadpoles and fishes [33]; and traps baited with these chemicals repel rather than attract the tadpoles of native frogs (above). Fortuitously, then, the substance that attracts toad tadpoles repels most native taxa. Any invasive-species control programme also needs to consider the ethical issues associated with killing animals [38], but most members of the general public are likely to find fewer ethical problems with killing larvae than with killing adult anurans.

Previous attempts to control invasive cane toads have focused on the terrestrial (post-larval) stages of the toads' life history [17,18]. That focus reflects the idea that density-dependent intraspecific competition is intense during larval life, so that removing a proportion of larvae may increase the rates of survival and growth of the remaining animals—and thus, have little net impact on eventual total recruitment from that cohort [39]. Putatively, density-dependence is less marked in terrestrial phases, because dispersal across the landscape reduces rates of intraspecific encounter. However, empirical evidence on the magnitude of these density-dependent effects is weak. For example, post-metamorphic toads compete strongly when dry conditions restrict them to the margins of natal waterbodies [40]; these conditions also facilitate cannibalism and parasite transfer [41,42]. Similarly, the long tropical dry-season concentrates adult toads in moist habitat patches (often, near buildings) for most of the year, creating competition for food [14]. Estimates of density-dependent effects within the larval stage are based on simplified enclosure experiments that may fail to mimic competitive interactions in natural waterbodies [43]. Putative ontogenetic shifts in the degree of density-dependence in toad populations [39] thus remain speculative.

Two factors suggest that control efforts targeted at larval toads might be more effective than heretofore assumed. First, cane toads spawn in a small and predictable subset of locally available waterbodies [44,45], whereas post-metamorphic stages are highly mobile and under moist conditions, can be widely dispersed across the landscape [40,46]. The larval toads' restriction to a few waterbodies means that control efforts at those sites can target an entire age-class within the local toad population. Second, our data suggest that we largely eradicated the toad tadpoles from waterbodies with a few days' trapping. Competitive release of survivors is unimportant if none survive. Even if some tadpoles do survive the trapping, those survivors are likely to be at heightened risk from predatory invertebrates (dytiscid beetles, belostomatid bugs [16,47]) because overall food supply for those predators has been reduced. Additionally, vertebrate predators such as fishes and frogs do not learn to avoid the (mildly toxic) toad tadpoles if these larvae are rare relative to palatable native tadpoles [48]. Hence, the reduction in toad tadpole numbers that we can achieve through trapping may result in minimal (or no) toad recruitment from at least some waterbodies.

In practice, how can we most effectively implement this new approach to cane-toad control? The technology to obtain toxin and build traps is simple, and well suited to implementation by the community groups that have been formed to combat the toad invasion [17,18]. The dangers of human exposure to toad toxins mean that toxin collection should be done by people who have been trained in safe procedures and are aware of the risks posed by these toxins. Future research could usefully explore ways to embed the toxin in a matrix that simultaneously prolongs its useful life as a bait (because it is released more slowly into the water) and renders it less easily ingestible by children or domestic pets. Also, future studies could search for less toxic components of the ‘cannibal attractant’ signal. For maximal effectiveness, we should combine toad tadpole eradication with existing methods for removing post-larval toads (hand-collecting and trapping adults); and of course, those ‘toad-busting’ activities provide a ready source of the toxin needed for toad-tadpole-trapping.

The continuing attractiveness of toxins for at least 3 days, combined with previous studies showing that the parotoid contents remain toxic for several months after a toad's death [32] suggest that toxin-baited traps may remain effective for long periods without bait replenishment (especially if the toxin is encased within a slow-release substrate). If so, a single deployment at the beginning of the toad's annual breeding season (which is concentrated in the brief wet-season [20]) might be enough to prevent toad recruitment from a given waterbody. Because the trap components are cheap and easy to assemble, and the bait is freely available in any area containing invasive toads, the only significant costs are for labour. Community concern about cane toad impacts means that free (volunteer) labour will probably be available, so the overall costs of deploying this new methodology would be far lower than for most control programmes targeted at feral pests [49].

Many invaders are taxa that attain high densities, often in disturbed sites where few other taxa occur [15]; these attributes may impose strong selection for an ability to locate and compete with rival conspecifics. The specific traits involved will vary widely: from allelopathy in the roots of weedy plant species [50,51] through to pheromonal suppression of reproduction in rodents [9]. More generally, ‘weed’ species often may possess intraspecific communication systems that can offer opportunities for invasive-species control. Entomologists have used pheromonal baits and lures for many years in the successful control of insect pests [52–54], and invasive mammal control has been facilitated by the use of ‘Judas goats’ [55]. Our results suggest that similar approaches hold great promise for the targeted control of invasive amphibians.
